# Mechanistic insights into sonication-assisted cold plasma treatments for improved microbial decontamination and quality maintenance in perishable foods^☆^

**DOI:** 10.1016/j.ultsonch.2026.107807

**Published:** 2026-03-03

**Authors:** Vinay Kumar Pandey, Pinku Chandra Nath, Shailendra Thapliyal, Ayaz Mukarram Shaikh

**Affiliations:** a Department of Food Science and Technology, Graphic Era (Deemed to be University), Dehradun, Uttarakhand, India - 248002; b Department of Bioengineering, School of Engineering and Architecture, Techno India University, Agartala 799004, Tripura, India; c Uttaranchal Institute of Technology, Uttaranchal University, Dehradun 248007, Uttarakhand, India; d Faculty of Agriculture, Food Science and Environmental Management, Institute of Food Science, University of Debrecen, Debrecen 4032, Hungary

**Keywords:** Sonication-assisted cold plasma, Non-thermal, Food preservation, Microbial inactivation, Shelf-life extension

## Abstract

Food preservation technologies are advancing towards the production of safe, minimally processed, and nutritious food products. Cold plasma (CP) is an emerging non-thermal technology that has received considerable attention for its potential application in decontaminating food from pathogenic and spoilage microorganisms with minimal damage to quality. Sonication, another acoustic cavitation-based technique, also provides similar synergistic advantages, such as mass transfer enhancement and disintegration of microbial membranes with enhanced exposure to reactive species. Sonication-assisted cold plasma (SACP), a combination of sonication and cold plasma, is a promising advancement in the preservation of food by enhancing the effects of microbial inactivation and shelf-life extension at lower energy input and treatment time. As well as its application for enzyme inactivation, bioactive retention, and packaging decontamination, the literature that is now accessible provides support for its potential use in fruits, vegetables, dairy products, and liquid foods. This review evaluates the fundamental concepts of sonication-assisted plasma activity. Additionally, the comparative advantages of this technology in comparison to stand-alone technologies are discussed, and the practical uses of this technology in food processing are highlighted.

## 1 Introduction

Food loss and waste are among the biggest global challenges with significant impacts on food security, the economy, and the environment. Post-harvest losses, particularly for perishable products (fruits, vegetables, dairy, and fish) worldwide, are approx. 20–40%, while country-level data and supply chain effectiveness [1]. These losses are mainly incurred by microbial spoilage, enzymatic hydrolysis, oxidative rancidity and improper storage of food along with reduced safety and nutrition of the food, wastage of resources and emission of harmful greenhouse gases [2]. Such losses have been compensated for by traditional methods of preservation, such as heat treatment, freezing, and chemical additives. Although proven to be effective in extending shelf-life and controlling pathogens, these methods usually cause unattractive changes in sensory qualities, nutritional value, and bioactive components, making them less acceptable among health-conscious consumers demanding fresh-like minimally processed foods. Thus, interest in non-thermal preservation technologies capable of preserving food quality and safety while extending shelf-life [3].

Non-thermal approaches represent a frontier in food preservation that combines microbial and enzymatic inactivation with ambient or near-ambient conditions to preserve more heat-sensitive nutrients and functional ingredients [4]. High-pressure processing, pulsed electric fields, UV radiation, and cold plasma (CP) have been investigated in recent years, demonstrating varying levels of efficacy based on the food matrix type. Among these, CP has demonstrated efficacy as a promising instrument due to its targeted action and multifaceted characteristics. CP is a partially ionized gas comprising reactive species, including reactive oxygen and nitrogen species, electrons, ions, and UV photons, capable of damaging microbial cell membranes, enzymes, and nucleic acid structures without significantly elevating food temperature [5]. This non-thermal approach has been successfully adopted for fresh fruits and vegetables, juices, dairy products, and packaged foods for surface cleansing and shelf-life extension. Nonetheless, due to constraints in penetration depth, uniformity of treatment, and scalability, it remains underdeveloped and not extensively utilized in the commercial food sector [6].

Another non-thermal procedure, sonication, is an emerging technology in the food industry because of its positive effects on mass transfer, extraction, and microorganism inactivation [5]. Ultrasonic vibration can cause intense compression and rarefaction at ultrasonic frequencies in liquids to create violent cavitation collapse, leading to localized high temperatures, pressures, and shear forces [7]. These effects can damage the microbial cell wall, stimulate intracellular component release, and accelerate the premeating speed of the sterilant, which in turn increases the effectiveness of preservation [8].

Sonication-assisted cold plasma (SACP) has recently been reported as an innovative method for overcoming the limitations of each technique [9]. Mechanical cavitation due to sonication and the reactive species generated from CP make this hybrid method a promising alternative for microbial inactivation by achieving higher yields of reactive species and reduction in treatment time [10]. More interestingly, the synergistic effect between ultrasonic waves and reactive species in plasma would help to decompose bacterial cell structures more effectively than ultrasound/plasma, which would be beneficial for decontamination and preservation [11]. SACP also showed the similar potential in retaining sensory and nutrition quality of food products as two non-thermo approaches which contribute to less heat damage of vitamins, pigments and bioactive constituents [12]. The synergistic use of DBD plasma, ultrasound, and thermal processing is very important for breaking down bacterial cell structures effectively. DBD plasma creates reactive species that harm cell membranes, proteins, and nucleic acids. Ultrasound, on the other hand, causes cavitation effects that make membranes more permeable and cause mechanical disruption. Thermal processing makes cells even weaker by changing the shape of proteins and making metabolic processes less effective. When used together, these treatments work together to speed up the breaking of membranes, the leaking of intracellular fluids, and the collapse of bacterial cells. This combined method works better at killing microbes than separate treatments, showing that it could be a good way to keep food safe and fresh.

A substantial reduction in surface microorganisms is observed as a result of fresh-cut fruits and vegetables with satisfactory texture, colour, and antioxidant content preservation [13]. SACP also exhibits anti-spoilage and pathogenic bacterial activities in liquid foods such as juice and dairy drinks without spoiling the flavour of the respective food or compromising their bioactivity [14]. Furthermore, the technology demonstrated effective enzyme inactivation capabilities, a crucial factor for preserving perishable goods. Additionally, novel applications may be devised, including the disinfection of food packaging materials, alongside the integration of hurdle technology and its implementation in industrial scaling processes [15]. These advantages support the suitability of SACP for addressing the present need for food conservation, particularly consumer requirements for the presence of safe, fresh-like, and minimally processed products [16].

The increasing demand for safe, high-quality, and sustainable food has driven the development of new non-thermal preservation technologies. SACP is a potential solution that combines the advantages of microbial inactivation enhancement with maintenance of sensory and nutritional qualities at both the laboratory and industrial levels [17]. By utilizing the advantages of cavitation effects and reactive species, SACP has surpassed several common issues with traditional CP treatment or sonication, suggesting that it has great potential for use in food preservation [18]. Consequently, researchers have commenced investigations into synergistic methodologies that amalgamate various non-thermal interventions. One such approach is the SACP, which unites the physical forces generated by acoustic cavitation with the chemical reactivity of plasma-derived species. This integration augments the transport of reactive molecules into food matrices, amplifies the efficacy of microbial and enzymatic inactivation, and facilitates the preservation of nutritional and sensory attributes. This review makes an effort to provide a comprehensive overview of the mechanisms, benefits, and applications of the SACP approach. In addition, it speculates on recent advances, challenges, and potential future trends for the utilization of this technology in the food sector for commercial purposes.

## 2 Principles of sonication-assisted cold plasma treatments

SACP is an emerging nonthermal method for food preservation, which combines sonication and the radicals produced by CP to inactivate microorganisms and enzymes and maintain the quality of food. The synergistic mechanisms of the technology have advantages over traditional processing approaches, with low thermo-chemical degradation, which is minimized.

### 2.1 Fundamentals of sonication

Sonication may be defined as a process using high-frequency sound(s), which refers to anything above the threshold of human hearing, around 20 kHz to 100 MHz, applied in a liquid or gas medium for various purposes [19]. These cycles create microscopic cavitation bubbles in the medium. The violent collapse of these bubbles results in the local production of high energy, leading to extreme temperature (∼5000 K) and pressure (∼1000 atm) states in the microzones [20]. This is called acoustic cavitation, and is the basic principle of sonication. In food preservation, cavitation involves mechanical, chemical, and thermal effects that assist microbial inactivation. When bubbles implode, they create microjets and shockwaves of energy that tear through the microbial cell walls and membranes, causing substances to leak into and microorganisms to die. At the same time, localized high temperatures and free radical creation are generated in microbial cells by sonication, which lack intracellular enzymatic systems for repair mechanisms resulting in DNA, protein, and enzyme injury. Sonication is an excellent non-thermal technology for the reduction of pathogenic and spoilage microorganisms in liquid foods, emulsions, and surface-processed solid food pathogenic and spoilage microorganism reduction [21], [22].

In addition to the bactericidal effect, sonication induces mass transfer processes, which help in the penetration of antimicrobial agents or reactive species into foods. It can enhance the extraction process of bioactive compounds, homogenize dispersions, and support emulsification; therefore, it is highly suitable for preservation as well as functional foods [23]. In addition, sonication is an energy-saving process that is eco-friendly because it reduces the cost of chemical preservatives and accelerates at room temperature, retaining heat-sensitive nutrients and organoleptic properties [24].

### 2.2 Basics of cold plasma (ionized gases, reactive species formation)

CP is a partially ionized gas of electrons, ions, neutral molecules and reactive species that has antibacterial properties without affecting the quality of food [25]. In contrast to thermal plasmas, CP is at or close to room temperature (approximately 40 °C); therefore, it is appropriate for use in heat-sensitive food systems [26]. During ionization, energy is delivered to a carrier gas (e.g., air, oxygen, nitrogen, or noble gases) via dielectric barrier discharge (DBD), corona discharge, or plasma jets, leading to a complex mixture of reactive oxygen species (ROS), reactive nitrogen species (RNS), ultraviolet photons, electrons, and charged particles [27].

The antimicrobial activity of these CP’s originates from oxidative stress caused by ROS (e.g., ozone, singlet oxygen, and hydroxyl radicals) and RNS (e.g., nitric oxide and peroxynitrite) [28]. These reactive species may react with the surfaces of microbial cells through lipid peroxidation, protein denaturation, and DNA damage to result in cell death [29]. UV photons and charged particles play a role in nucleic acid breakage and membrane depolarization, which increases the total effect of inactivation [30]. A low thermal load also maintains sensorial and nutritional characteristics, including colour, flavour, and bioactive compounds, which are frequently detrimentally affected by classical thermal treatments [31].

CP can be utilized either directly or indirectly. For direct plasma treatments, the food product is irradiated with plasma discharge to optimize the contact between it and the reactive species. Indirect treatments are applied to a medium (e.g., gas, water, or package surface), and the product is indirectly exposed to the plasma [24]. Both methods allow the decontamination of surfaces, inactivation of enzymes, and even destruction of pesticide residues. However, CP can have limitations in acting on complex food matrices with uneven surfaces and dense structures because of the limitation of reactive species diffusion lengths, which are generally confined to shallow layers [32].

### 2.3 Synergistic effects of combining sonication and cold plasma

CP coupled sonication integrates the mechanisms of both technologies, with complementary and synergistic effects for microbial inactivation and food preservation [33]. Sonication generates cavitation-induced mechanical forces, microjets, and localized elevated temperatures as they are CP produce ROS, RNS, UV photons and charged particles exerting oxidative and lethal structural effects on microbial cells [34]. When used in a simultaneous or sequential manner, sonication can enhance the depth of penetration of plasma-produced reactive species through microbial biofilms and into uneven surfaces or dense food substrates over plasma treatment alone [35].

Mechanistically, the mechanical effect and shear forces from ultrasonic cavitation induce transient formation of pores in microbial cell membranes that permit more efficient access to ROS and RNS, which attack intracellular sites such as nucleic acids, enzymes, and proteins [36]. This combined effect allows for the inactivation of microorganisms at a high rate, frequently exceeding the sum of individual treatments. Sonication causes local turbulence and post-microstreaming, which greatly improve the movement of mass through liquids and across food surfaces. These physical effects help reactive species spread quickly and evenly, which helps them get into complex food matrices and boundary layers more easily. As a result, the reactive molecules made during treatment are more evenly delivered to microbial cells and surface irregularities, which reduces untreated areas. This better way of moving things around makes it easier for reactive species to come into contact with target microorganisms, which makes microbial inactivation more effective. Also, consistent exposure reduces quality loss caused by uneven treatment, which makes the process more efficient and reliable. Sonication is a useful extra method for advanced food preservation strategies because it improves mixing and transport [37], [38]. The combination therapies utilizing SACP exceeded the mere aggregation of individual components. The inactivation of enzymes, a significant factor in postharvest deterioration, is expedited following the accumulation of mechanical and oxidative damage [39]. Moreover, the hybrid methodology results in decreased treatment duration and energy consumption during processing, as the utilization of either technique amplifies the efficacy of the other. The sensory and nutritional attributes are more effectively preserved compared to prolonged single treatments, encompassing the retention of vitamins, pigments, antioxidants, and vital substances [40].

### 2.4 Comparison with conventional preservation methods

Traditional methods of food preservation such as heating, refrigeration, freezing and chemical preservations are commonly used to increase the shelf life and maintain microbial safety [41]. Physical treatments such as pasteurization and sterilization use heat to inactivate microorganisms and enzymes. Although these processes are successful, they often result in unwanted quality changes in texture, flavour, colour, and nutritional content, especially when handling heat-sensitive foods such as fresh fruits, juices, and dairy products [42]. Chemical preservatives such as sulphites, benzoates, and nitrites can inhibit the growth of microorganisms but are now being subjected to even more consumer rejection than in the past because of health concerns and regulatory guidelines. Refrigeration and freezing are effective at slowing microbial metabolism but not killing pathogens, they both must operate uninterruptedly with input of energy and storage facilities [43].

Non-thermal methods, such as sonication and CP, have been studied to address these limitations using low heat or chemical additives involved in microbial elimination. Sonication achieves this through cavitation-based mechanical and physical effects, whereas CP dismantles microbial cells through reactive species and UV photons [44]. In SACP, they also synergize, thereby enhancing the kill efficacy, homogeneous treatment distribution, and shortened process time [45]. Compared to thermal methods, SACP can help sustain the sensory and nutritional quality of end products by preserving vitamins, antioxidants, flavour compounds, and pigments. In addition, processing requires less water and energy than conventional processing, which is in accordance with the current food processing sustainability requirements [46].

Comparisons show that SACP often attains the same levels of microbial inactivation as thermal pasteurization, without destroying product quality. SACP-processed fresh-cut fruits and juices are highly effective in retaining texture, colour loss, and antioxidant activity relative to thermal pasteurization [47]. Moreover, SACP can be applied to food packaging and surfaces, which is a much wider application than existing technologies depending on direct heat or chemicals. Overall, sonication-supported CP is an emerging environmentally friendly technology that bridges efficacy and quality retention in modern food environments [48].

## 3 Mechanism of action

The biological action of SACP is mediated by the combined effects of cavitation-driven physical processes and the reactive species present in the plasma. Oxidative stress, peroxidation of cell membranes, and chemical alterations have been attributed to these conversions in microbial cells, as well as food structure [49].

### 3.1 Generation of reactive oxygen and nitrogen species (RONS)

One of the primary drivers of CP treatment is RONS, including ozone, hydroxyl radicals, hydrogen peroxide, singlet oxygen, nitric oxide, nitrogen dioxide, and peroxynitrite [50]. Ionized carrier gases such as oxygen, air, or nitrogen are the most reactive species generated by plasma discharge. For example, oxygen plasma can create ROS by electron impact dissociation, and nitrogen plasma and RNS via molecular excitation and recombination [51]. The presence of both gases has the potential for synergistic action, resulting in a rich blend of RONS with broad-spectrum antimicrobial activities. Oxidative stress resulting from ROS and reactive nitrogen species (RNS) has been associated with various diseases. On microbial surfaces, they initiate lipid peroxidation, which increases membrane fluidity, permeability transition, pore formation, and ultimately, lysis [52]. These free radicals also damage proteins, enzymes, and nucleotides (nucleic acids) present in cells, thereby interfering with vital metabolic processes inside the cells. Crucially, the simultaneous existence of short-lifetime species (e.g., ^•^OH and ^1^O_2_) vs. long-lifetime O_3_ or H_2_O_2_ offers sudden and prolonged sterilization activities [53].

**Fig. 1:**
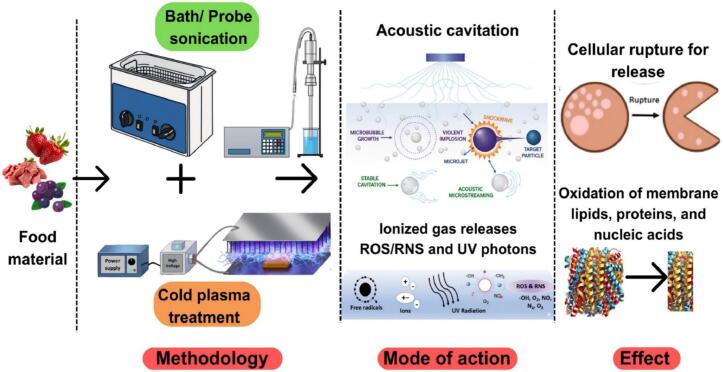
Synergistic antimicrobial mechanism of sonication and cold plasma treatments on food surfaces. Fig. 1 is reproduced with permission (copyright © 2026 Elsevier B.V., Amsterdam, the Netherlands) from [12].

The accessibility and distribution of RONS were improved by sonication under these conditions. Microstreaming and turbulence (in both the gas phase as well as the fluid phase) are enhanced by ultrasonic cavitation, promoting a more effective penetration of reactive species into biofilms, food matrices or microbial cells [54]. Furthermore, the point-collapse of cavitation bubbles gives rise to further free radicals, for example, ^•^OH, in addition to plasma-generated RONS, thereby accelerating oxidative stress. This combinatorial production and dissemination of RONS is responsible for the enhanced microbicidal potential that the SACP design holds over its monotherapy counterpart [55]. Fig. 1 schematically shows the synergistic inactivation of microorganisms adhering to food surfaces induced by combined ultrasonication and cold plasma treatment. During sonication, microbial cell walls are mechanically disturbed, pores (sonoporation) are produced, and the penetration of reactive oxygen and nitrogen species (RONS) increases, whereas with cold plasma lipid peroxidation, UV-induced damage to lipids, proteins, and DNA is observed, along with cytoplasmic acidification. The combination of the two non-thermal technologies could produce synergistic effects, such as improved rate of cell death, more serious membrane breakage, and higher microbial inactivation efficiency, and lead to better food safety and high quality.

### 3.2 Electropore formation and electroporpermeabilization in microbial cells

Electroporation refers to the creation of pores in the membranes of microorganisms, which may be either transient or permanent, due to exposure to high electric field strengths and/or reactive conditions. In CP processing, charged particles and electrostatic fields exert a localized effect on microbial cells by inducing membrane depolarization, resulting in the destabilization of the lipid bilayer [56]. This process facilitates pore formation, thereby enhancing membrane permeability and compromising cellular integrity. Sonication works synergistically to generate mechanical shear forces and shock waves from the collapse of cavitation bubbles. These forces weaken cell walls and membranes, making them more susceptible to disruption by plasma-induced electroporation [57]. The ephemeral and permeable pores enable the effective traversal of the cytoplasmic membrane by reactive oxygen and nitrogen species (RONS) and other antimicrobial agents, thereby enhancing intracellular oxidative stress and damage. Electroporation may remain reversible when the harm is minimal; however, the cumulative stress induced by SACP could lead to permanent membrane rupture and cellular demise [58]. The creation of electropores during treatment markedly disturbs the internal ionic equilibrium of microbial cells, resulting in the efflux of critical intracellular components such as ATP, nucleotides, and crucial ions. This loss undermines cellular metabolism and energy equilibrium, inducing significant physiological stress. The unregulated transport of solutes across the membrane induces osmotic imbalance, leading to membrane swelling, structural damage, and ultimately cell rupture, which hastens microbial inactivation. The structural variations across bacterial groupings affect their vulnerability to this mechanism. Gram-negative bacteria, distinguished by a thinner peptidoglycan coating and comparatively greater membrane fluidity, are typically more susceptible to damage generated by electroporation. Conversely, gram-positive bacteria have a more robust peptidoglycan matrix that offers partial defense against membrane permeabilization. Under optimum SACP treatment conditions, both gram-negative and gram-positive bacteria can be efficiently eradicated. These findings underscore the extensive antibacterial efficacy of SACP and its capacity to surmount intrinsic variations in bacterial cell wall structure via regulated electrical and plasma-induced mechanisms [59].

### 3.3 Role of hydroxyl free radicals in oxidative stress and microbial inactivation

During sonication and CP, free radicals, including hydroxyl radicals (^•^OH), are the most powerful oxidizing agents. In sonication, followed by the pyrolysis of water vapor, collapsing cavitation bubbles and dissociation from water and oxygen molecules under electron impact are formed. SACP systems can promote the accumulation of ^•^OH, which causes a higher oxidative stress in microbial ecosystems [60]. Hydroxyl radicals are very short-lived but extremely reactive, and can react with virtually all biomolecules near the site of their generation. In microbial cells, ^•^OH is involved in the lipid peroxidation of their membranes, leading to interference with the bilayer structure as well as cytoplasmic leakage [61]. In cells, ^•^OH interacts with nucleic acids, causing chain breaks, modified bases, and crosslinks, which inhibit replication and transcription. Proteome and hydrolase activities are also affected by oxidation processes, leading to the denaturation, aggregation, or inactivation of some proteins [62].

The remarkable property of ^•^OH radicals is their ability to cause permanent, non-specific damage to microorganisms that have limited repair capabilities. Hydroxyl radicals are so reactive that they react directly with the solute molecule at a diffusion-controlled rate, rather than first reacting to form more selective toxic compounds, such as hydrogen peroxide [63]. In SACP, the simultaneous production of ^•^OH by cavitation and plasma causes locally confined microenvironments in which the oxidative stress level is higher than that withstood by microbial catalases or superoxide dismutase [64].

### 3.4 Molecular and biochemical interactions with enzymes and cell membranes

In addition to microbial inactivation, SACP affects enzymes and cellular macromolecules, which provides more effective food preservation. During storage, undesirable changes in colour, flavor, and texture also occur because of enzymes such oxidase (PPO), peroxidase (POD) and lipoxygenase (LOX) [37], [38]. Plasma-produced RONS and cell-sonication-generated radicals attack proteins directly from enzymes, oxidizing amino acid residues, breaking disulfide bridges, and modifying secondary and tertiary structures [65]. The mutator technique may result in the loss or diminishment of the catalyst function.

The sulfhydryl groups in cysteine residues can be altered by hydroxyl radicals and ozone, which can lead to conformational changes that interrupt the functioning of the enzyme. The oxidation of tyrosine and indole (tryptophan) residues is also a possibility, as it has the potential to cause the rupture of hydrophobic areas and the instability of the enzyme [66]. Sonication is responsible for the formation of shear pressures, which denature proteins and stretch out their original structure. This results in oxidatively sensitive groups being exposed to oxidation by plasma-derived molecules, which leads to the deactivation of biological enzymes [67]. The lipid bilayer is subjected to peroxidation as a result of the cofactor action of RONS and cavitation, which causes the cell membranes to be impacted as well. Since this has an influence on the fluidity, permeability, and receptor-effector coupling of the membrane, it leads to a reduction in the amount of nutrient intake and microbial communication (quorum sensing). In plant and animal cells, the oxidation of membranes slows down the pace of enzyme activity and metabolic rates, which in turn slows down the rate at which food systems become obsolete [68].

### 3.5 Impact on food microstructure and biochemical stability

In addition to inactivating microorganisms and enzymes, SACP has an effect on the quality of food, including alterations in the microstructure and the biochemical stability of the food. Cavitation forces are exerted by sonication, which can affect the integrity of the cell wall, disintegrate starch granules, or break macromolecules. This results in an improvement in the extractability of bioactive chemicals and in the homogeneity of the sample [69]. In the meantime, CP has an effect on the surfaces and constituents of food, which can result in cross-linking, oxidation, or polymerization respectively, depending on the matrix nature and the parameters of the process [66].

The fresh SACP product preserves firmness and maintains colour by inactivating softening and browning enzymes, protecting pectin from degradation, and reducing the loss of pigments [70]. The beneficial effects of sonication on the reduction of particle size and increase in emulsion stability are similar to those reported in dairy and liquid systems, whereas plasma modifies the proteins on the surface, thereby improving foaming and emulsifying properties [71]. In combination, these properties lead to an enhanced texture and sensory characteristics.

Biochemically, it is hypothesized that the combined treatment saves antioxidants, vitamins, and phenolic compounds as a consequence of being applied for less time in a high-energy plasma atmosphere [72]. However, despite the importance of oxidative stress in microbial killing, nutrient degradation can be minimized under well-tuned SACP conditions [73]. This balance between the efficiency of pathogen killing and nutrient retention makes SACP distinct from thermal processes, where heat-sensitive constituents are often denatured.

## 4 Benefits of sonication-assisted cold plasma treatments

In comparison to traditional treatments, the synergistic effects of sonication and CP have been found to offer a number of benefits, including an advantage in terms of the inactivation of microorganisms, as well as an advantage in terms of certain qualitative features [74]. The enzymatic activity of products is reduced by this innovative hybridization technique, which also helps to maintain the products' sensory qualities and bioactive substances while extending their shelf life.

### 4.1 Health-promoting compounds

SACP has the important advantage of preserving natural products that are usually lost during heat pasteurization and other thermal preservation processes, as well as vitamins, antioxidants, and polyphenols [75]. Antioxidants, including vitamin C, carotenoids, flavonoids and phenolic acids are important to the human body for dealing with oxidative stress and act as bioactive factors related to quality characteristics of functional foods [76]. Sensitive substances can be disassembled even with cold plasma if the treatment time or potency is high (owing to excessive oxidative stress). Sonication-mediated plasma prevents this by shortening the treatment time and promoting a uniform distribution of reactive species, thereby preventing the local overexposure of nutrients. Ultrasonication also facilitates the liberation of bound phenolics and antioxidants from cell walls and vacuole by cavitation which maximizes the availability of these compounds [77].

Vitamin C and phenolic losses in vitamin C and phenolic contents were significantly lower than those in the thermal pasteurization of fruit juices, leafy vegetables, and berries [78]. The antioxidant activity also increases as a result of the improved extractability of bioactive compounds by cavitation [79]. For instance, ultrasound-enhanced plasma treatment of citrus juice resulted in better ascorbic acid and total polyphenol retention when compared to conventional thermal treatment, in addition to microbiological safety [24]. Research on fruit juices and juice extracts of leafy vegetables and berries has shown very low loss of vitamin C and phenolics, as compared with that of thermal pasteurization when treated with SACP [80]. In some cases, antioxidant activity is increased by the improved extractability of bioactive substances that cause cavitation. For instance, ultrasound-assisted plasma treatment of citrus juices resulted in greater retention of total polyphenols and ascorbic acid than the conventional heat process, while remaining microbiologically safe.

### 4.2 Retention of freshness

To maintain the fresh-like quality of food, natural appearance (color, texture, and aroma) is one of the challenging aspects of food preservation. Traditional processes based on the application of heat can cause pigmentation breakdown, textural limpness and evaporation of volatile aroma components [37], [38]. SACP has a great advantage in terms of these freshness properties because it is performed under mild non-thermal conditions. These effects may be attributable to the fact that RONS generated by CP might inactivate some enzymes of browning and off-flavour, such as peroxidase and lipoxygenase [81]. Sonication also facilitates this effect, as discentered enzyme aggregates aid the homogeneous dispersion of RONS. For fresh-cut lettuce and strawberries, SACP treatments helped to maintain the colourful chlorophyll and anthocyanin pigments, leading to stable colour levels during storage [82]. Carotenoids, which are responsible for the colour of carrot juice, as well as lycopene in tomatoes, were largely preserved after treatment.

Texture maintenance is due to the slight thermal effect of SACP with pasteurization, which is often associated with pectin breakdown and tissue softening [83]. Sonication also helps homogenize the internal cellular structures, deflating rather than softening when not in significant quantities. Apples and cucumbers studies have illustrated that SACP is still hard but reduces microbial load significantly [68]. Ester and terpene volatiles, in particular, are susceptible to heat but are relatively stable under SACP. Natural flavour profiles were preserved in fruit juices, and there was less volatile loss in dairy media compared to heat pasteurization [84]. In addition, the reduced steeping time reduced the oxidative destruction of flavour compounds.

### 4.3 Shelf-life extension

The primary purpose of preservation technologies is to increase shelf life by retarding microbial spoilage and deterioration of quality. This is accomplished by the synergistic neutralization of bacteria, yeasts, and moulds, and stabilization of biochemical and sensory characteristics. For example, in SACP-coated fresh-cut fruits, a reduction in mesophilic bacteria and fungi of more than 2–4 log was observed, which extended shelf life for 7–10 days during refrigerated storage [37], [38]. Microbial counts in the dairy drinks were maintained below the spoilage limits set for such products throughout the long storage period, without changing the nutritional and sensory properties. Fresh juices and smoothies demonstrated extended microbial stability coupled with antioxidant preservation leading to product freshness during storage [85].

**Fig. 2:**
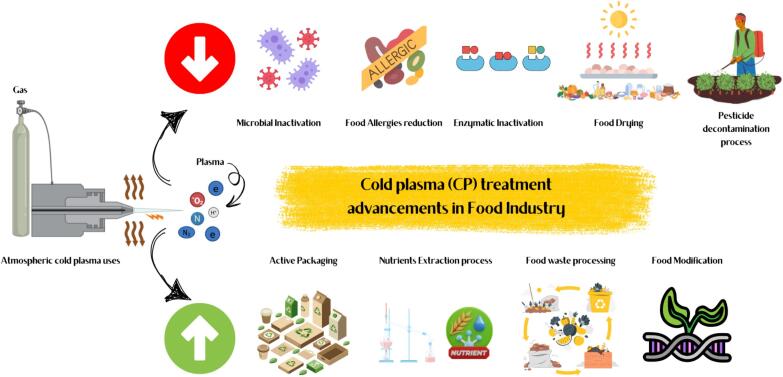
Advancements of cold plasma treatment in food industry.

In addition to bacterial control, SACP retards enzymatic and oxidative reactions that contribute to the loss of food quality. Disruption of the enzyme structure due to sonication and the change in its activity centre or breakdown of phenolic substrates involved in browning reactions are exhausted by plasma-induced RONS [86]. Together, these processes suppress enzymatic browning, lipid oxidation, and textural breakdown, which are the key contributors to product spoilage [83]. Accordingly, foods treated with the composition possess preserved colour and odour as well as sample firmness during extended periods of storage which adds to their appeal in the ultimate marketplace [87]. Fig. 2 illustrates diverse applications such as microbial and enzymatic inactivation, food allergy reduction, pesticide decontamination, food drying, active packaging, nutrient extraction, food waste processing, and food modification, highlighting its role in enhancing food safety, quality, and sustainability.

## 5 Applications in food industry

### 5.1 Fresh-cut fruits and vegetables

Fresh fruits and vegetables are among the most perishable food products because of their high moisture content, enzymatic activity, and microbial infestation [13]. Postharvest losses continue to be a serious challenge worldwide, and spoilage during transport and storage remains an important source of food waste. SACP treatment is a promising technology to overcome these challenges in fresh produce, as it can reduce microorganism loading and delay waste degradation with extended shelf life [88]. The antimicrobial effect of SACP is particularly beneficial for fresh-cut or minimally processed fruits and vegetables, including, but not limited to, fresh-cut apples, strawberries, lettuce and spinach which are susceptible to rapid microbial growth [89]. RONS produced during plasma operation are aided by sonication-driven cavitation to inactivate bacteria, yeasts, and moulds on produce surfaces without the deposition of chemically based residues [90]. This multitarget effect, in addition to decreasing the risk by foodborne pathogens, like *E. coli* O157:H7, *L. monocytogenes* and *Salmonella* spp., reduces spoilage microorganisms' counts resulting in an extended shelf life of these products [91].

Some promising findings have emerged from the experimental research. For instance, strawberries treated with SACP displayed significantly improved firmness and lower total microbial counts than unthreaded controls during storage [90]. SACP-treated whole leafy greens also had a higher chlorophyll content and visual freshness during extended refrigerated storage [92]. These findings reveal the promise of SACP in mitigating postharvest losses and meeting the demand for high-quality produce, locally as well as internationally. According to Bao et al. [93], CP treatment increased the extraction yields of polyphenols from tomato by-products by 10% [93]. Furthermore, sonication has been extensively utilized as an effective extraction technique in prior research. The yields of selected compounds can be markedly enhanced with sonication aid, attributed to the cavitation effect and the stimulation of mass transfer by ultrasound [94]. Integrating CP with sonication enhances extraction efficiency, hence expanding the application potential of innovative food processing technologies.

Polyphenol oxidase (PPO) is a critical enzyme that catalyses the oxidation of phenolic substrates to quinones, followed by their polymerization to brown pigments. Traditional methods to control PPO, such as sulphate treatment or heating blanching, would either leave chemical residues or damage texture and nutrition. SACP provides a residue free, nonthermal alternative for inactivation of PPO [95]. CP also produces RONS (e.g., ozone, hydroxyl radicals, and nitric oxide), which can oxidize amino acid residues important for PPO activity. The cysteine, tyrosine, and histidine residues in the active site of the enzyme are subject to oxidative modification, which causes changes in its conformation and, consequently, its less efficient functioning [24]. Sonication magnifies the effect of opening up enzyme structures via cavitation-induced shear forces, thereby exposing the active sites to plasma-derived RONS for site-specific inactivation. Significant inactivation of PPO was observed on apples, bananas, and potatoes by SACP treatment which caused a retardation in browning during storage yet did not affect the texture and flavour [96]. In addition, the 2-fold action decreases the time period and strength of treatment compared to single plasma or ultrasound treatments, which decreases undesired oxidative reactions in food matrices.

### 5.2 Dairy products

Milk, cheese, yogurt, and cream are nutritious but highly perishable because of their richness in proteins, fats, and sugars, which provide an environment for the rapid growth of spoilage and pathogenic microorganisms [85]. Pasteurization and ultra-high-temperature (UHT) processing are routine preservation processes used to eliminate pathogens and extend the shelf life while maintaining safety, however, heat-driven techniques can lead to unwanted flavour, texture and nutritional changes [97]. Non thermal techniques, such as SACP, can potentially promote microbial safety and shelf life of dairy products without significant changes in sensory and nutritional quality [98].

The synergistic effect of ultrasound and plasma-induced reactive species is a determining factor in the inactivation of microorganisms during treatment with milk matrices [99]. The microbial cell envelope is destroyed by ultrasound-induced cavitation, and RONS derived from cold plasma cause oxidative damage to crucial biomolecules, including DNA and proteins. This combined action reduces spoilage of pathogenic organisms (such as *Staphylococcus aureus, E. coli* and *Listeria monocytogenes*) [100]. Crucially, SACP accomplishes this without incurring significant heat, thereby protecting heat-sensitive bioactive ingredients, including vitamins, immunoglobulins, and probiotics [101]. Physicochemical quality is another important aspect of dairy preservation, and the best results have been obtained in PM preservation after SACP. To date, it has been reported that treated milk could maintain the natural flavour, colour, and viscosity with only slight changes in fat globule destruction and protein conformation. Lipid oxidation, a key mechanism of off-flavour development in dairy products, is regulated by tailoring duration of plasma exposure and intensity with sonication [102]. Moreover, SACP can retard enzymatic action during spoilage, thereby prolonging its freshness and stability during storage.

### 5.3 Meat and poultry products

Meat and poultry are particularly perishable owing to their high-water activity, nutrient content, susceptibility to microbial spoilage, and oxidative deterioration. *Pseudomonas* spp., as well as pathogenic bacteria such as *Salmonella, Listeria monocytogenes* and *E. coli* O157:H7 are most common in this group [103]. Procedures such as refrigeration, freezing, and the addition of chemicals are not effective in providing a permanent solution to the problems of microbial safety and oxidative stability [104]. On the other hand, SACP is emerging as a new treatment method that enhances microbial inactivation and oxidative spoilage inhibition, allowing extended shelf life of meat and poultry with minimally affected sensory attributes [105].

The antibacterial mechanism of SACP in meat systems is ascribed to the production of RONS during plasma treatment, which in turn promotes mechanical effects through sonication. Sonication promotes cavitation to induce the DCD process, reducing membrane integrity for microorganisms and biofilm protection which in turn permitted further penetration of plasma-produced RONS [106]. This crosstalk introduces wounds inflicted by MAC and oxidative stress into microorganisms, which reduces viable counts to a safer value. Therefore, SACP has been demonstrated to reduce surface contamination in raw food such as portioned poultry cuts, ground beef and ready-to-eat meat items with high efficacy value for protection of foodborne pathogens [107]. In addition to microbial safety, SACP also contributes to quality maintenance in meat and poultry products. The off-flavour, discoloration, and loss of nutrition of meat are mainly due to lipid oxidation. By adjusting the cure methods, SACP could inhibit oxidation reactions and maintain good qualities, such as meat colour stability, tenderness, and juiciness. The cavitation resulting from sonication also promotes homogeneous exposure of plasma, and thus even treatment throughout complex surfaces of meat [108].

### 5.4 Seafood products

Seafood, such as fish, shrimp, mollusks, and crustaceans, are among the most perishable food types because of their high protein and unsaturated lipid contents, neutral pH, and rich free amino acids that favor microbial growth and oxidative spoilage [109]. Postharvest losses in seafood are especially serious, as damage occurs shortly after they are harvested or in a matter of days under ambient temperature. Conventional preservation techniques for prolonging shelf life of whole fish and fish cuts. The conventional techniques including icing, freezing and application of additives can arrest spoilage however may not always ensure safety and quality particularly during long term storage and transportation [110]. SACP has been found to be a very promising new technology that can make the nonthermal and residueless effects of CP on the inactivation and quality maintenance of seafood.

The synergistic effect of ultrasound and plasma-derived RONS shows efficient inactivation of spoilage microorganisms and seafood-associated food-borne pathogens, such as *Vibrio parahaemolyticus*, *Listeria monocytogenes*, and *Salmonella* spp., which triggers cavitation that ruptures biofilms and weakens microbial cell walls, allowing better permeation of plasma-generated RONS, leading to oxidative disturbance in microbial DNA, proteins, and membranes [111]. This dual effect considerably reduces the initial microbial load and retards the regrowth of the microflora, consequently prolonging both the safety and shelf life of seafood products. Apart from microbiological inactivation, SACP also deals with quality concerns related to lipid oxidation, as it is one of the major factors of rancidity and the development of off-flavor compounds in seafood [112]. Plasma treatment results in the production of RONS, increasing oxidation if too strong; however, when combined with sonication under appropriate conditions, it can inhibit excessive lipid degradation and improve microbiological safety [113]. Therefore, the color and aroma of the SACP-treated seafood were longer than those of the untreated control. In addition, texture preservation is promoted by slowing proteolytic enzyme activity, which causes softening during storage.

### 5.5 Bakery and cereal products

Bakery and cereal products, such as bread, cakes, biscuits, and ready-to-eat cereals, are consumed worldwide; however, they are affected by microbial spoilage during storage. Moulds, yeasts, and some bacteria are the most common causes of spoilage of these products, resulting in visible fungal growth on cheese surfaces [114]. Traditional preservation methods are mainly based on chemical additives, modified atmosphere packaging (MAP) and refrigeration. Nevertheless, increasing consumer demands for “clean label” products and foods with less additives have caused the interest in nonthermal technologies including SACP as a tool for shelf-life extension of bakery/cereal-based products [115].

SACP exhibits robust antibacterial activity against spoiling fungus and bacteria on bakery surfaces. Plasma-generated RONS can penetrate microbial cell walls by creating oxidative stress and causing cell death. Ultrasound amplifies this action by breaching protective barriers and homogenizing reactive particles on the porous and irregular surfaces of food [116]. This dual impact renders SACP especially appropriate for use in businesses producing breads, cakes, and pastries that have extensive surfaces vulnerable to mould infestations. Besides microbial eradication, SACP can affect the physicochemical stability of bread and cereal products [117]. Lipid oxidation in high-fat bakery products, such as cookies and cakes, would be diminished under optimum treatment settings. Moreover, starch retrogradation, a primary factor in bread staling, may be postponed by the structural alterations induced by sonication, thereby aiding in the maintenance of softness and texture. Plasma-induced changes can block the activity of enzymes responsible for quality deterioration, hence contributing to extended freshness.

### 5.6 Beverages

Beverages have a high potential for microbial contamination and quality deterioration owing to their high-water activity, nutrient composition, and frequently low pH levels. Traditional preservation techniques to prolong shelf life, such as pasteurization and the use of chemical preservatives, may result in sensory quality or loss of bioactive compounds (such as vitamins, polyphenols, and antioxidants) loss [118]. SACP is a nonthermal method that can improve the microbiological safety, nutritional qualities, and shelf life of beverages without creating significant changes in the sensory quality of the beverage. Ultrasound and RONS produced by plasma are both necessary components for the destruction of microorganisms in liquids. Cavitation is produced by ultrasonic vibrations, which results in the development of microbubbles. This microbubble generation causes the cell walls of microorganisms to rupture, which in turn releases active species into liquids [119]. Oxidative stress is caused by plasma treatment, damaging DNA, proteins, and lipids in spoilage microflora and pathogenic bacteria such as *E. coli* O157:H7, *Salmonella* spp., *Listeria monocytogenes* [120]. This partnership ensures the best micro-bacterial reduction, yet still the protection of heat-sensitive nutrients that would otherwise be altered by the heating process.

In addition to microbial safety, SACP preserves the functional and sensory attributes of the beverages. Enzymatic browning in fruit juice catalyzed by PPO and POD may be inhibited by enzyme inactivation to maintain natural color and flavour [121]. Similarly, lipid oxidation-induced off-flavor formation in plant-derived milk alternatives, such as soy and almond, with optimization of incubation conditions is diminished. Research has demonstrated that juices processed using SACP are richer in vitamin C, phenolic compounds, and antioxidant capacity than pasteurized products; therefore, this technology is particularly relevant for health-promoting beverages [122]. The applications of SACP treatment for food preservation are shown in Table 1.

**Table 1:** Applications of sonication-assisted cold plasma treatments in food preservation.

**Food Category**	**Associated Microorganisms**	**Application Purpose**	**Treatment Conditions**	**Findings**	**References**
Fresh-Cut Fruits (Apples, Strawberries, Lettuce)	*E. coli* O157:H7, *Listeria monocytogenes*, *Salmonella* spp.	Surface microbial decontamination and shelf-life extension	Sonication: 20 kHz, 200 W, 5–10 min; Cold plasma: 50 kV, 10 min, air as working gas	∼3–4 log CFU/g reduction; shelf-life extended to 10–12 days	[133]
Fruit Juices (Orange, Pomegranate, Apple)	*Salmonella enterica*, *E. coli*, spoilage yeasts	Non-thermal pasteurization and nutrient retention	Sonication: 24 kHz, 400 W, 10 min; Plasma: 60 kV, 8 min, argon gas	99.9% microbial inactivation; 90–95% vitamin C retention	[1]
Milk and Dairy (Raw milk, Yogurt)	*E. coli*, *Staphylococcus aureus*, *Listeria* spp.	Pathogen inactivation and quality preservation	Sonication: 20 kHz, 300 W, 5 min; Plasma: 70 kV, 10 min, helium	4–5 log CFU/mL reduction; minimal protein denaturation	[134]
Meat and Poultry (Chicken breast, Beef)	*Listeria monocytogenes*, *Campylobacter jejuni*, *Salmonella* spp.	Surface sterilization and spoilage delay	Sonication: 25 kHz, 250 W, 8 min; Plasma: 55 kV, 12 min, oxygen gas	2–3 log reduction; lipid oxidation reduced by ∼ 25%; shelf-life extended 5–7 days	[135]
Seafood (Shrimp, Salmon)	*Vibrio parahaemolyticus*, *Listeria*, *Pseudomonas* spp.	Pathogen reduction and oxidation control	Sonication: 20 kHz, 300 W, 7 min; Plasma: 50 kV, 8 min, air/argon mix	2–4 log reduction; delayed lipid oxidation; improved freshness	[111]
Cereals and Grains (Wheat, Rice)	Aspergillus flavus, *Penicillium* spp., *Fusarium* spp.	Decontamination and fungal/mycotoxin control	Sonication: 22 kHz, 200 W, 6 min; Plasma: 60 kV, 15 min, air plasma	∼80–90% fungal spore reduction; safe storage > 6 months	[136]
Ready-to-Eat Foods (Salads, Sandwiches)	*E. coli* O157:H7, *Listeria monocytogenes*, *Salmonella* spp.	Surface decontamination and safety assurance	Sonication: 20 kHz, 250 W, 5 min; Plasma: 55 kV, 10 min, nitrogen gas	2–3 log reduction; sensory attributes preserved	[129]
Cumin seeds (*Cuminum cyminum* L.)	*Aspergillus* spp., *Penicillium* spp., *Bacillus* spp., *and E. coli*	Reduce microbiological development, increase product safety, and preserve cumin seed quality, aroma, and medicinal components throughout storage.	Ultrasound-enhanced convective drying at 60, 120, and 180 W	Reduction in maximum drying time: 46.65%	[137]
Caraway seeds	*Aspergillus* spp., *Penicillium* spp., *Bacillus* spp., *Salmonella* spp., *and E. coli*	Ultrasound-assisted convective drying reduces microbiological contamination, increases drying efficiency, preserves caraway seeds' sensory characteristics and essential oil content, and improves storage stability and food safety by 31%.	Ultrasound (US) at 60, 120, and 180 W during convective drying at temperatures of 35, 45, and 55 °C	Drying duration decreased by 31%.	[138]

### 5.7 Ready-to-eat and convenience foods

Pre-cooked and convenience foods: Snacks, ready-to-eat meal solutions, ready-to-cook meal kits, and salad pack-consumers are continuing to transition towards pre-cooked and convenience meals both due to our changing lifestyles as well as the need for fast, safe, and healthy food options [123], [124]. Nevertheless, such products are often eaten as they are, and are therefore susceptible to microbial spoilage and degradation. Shelf life is limited by the growth of spoilage and pathogenic organisms such as *Listeria monocytogenes*, *Salmonella* spp., and *E. coli* O157:H7; thus, preservation becomes a major challenge [125].

SACP is a compelling non-thermal alternative for enhancing the safety, quality retention, and shelf-life of RTE foods while maintaining customer preference for minimally processed products [126]. The antibacterial action of SACP in ready-to-eat meals is fundamentally reliant on the synergy of ultrasound and plasma-derived reactive oxygen and nitrogen species. Ultrasound-induced cavitation breaks cell membranes and biofilms, facilitating the migration of reactive species to intricate food surfaces [127]. Subsequently, RONS catalyse the oxidation of microbial DNA, proteins, and membranes, resulting in the swift elimination of pathogens and spoilage microorganisms. This dual methodology is particularly advantageous for multi-component ready-to-eat foods, characterized by heterogeneous surfaces and microenvironments conducive to microbial survival [128]. SACP treatment has been shown to extend the refrigerated shelf life of ready-to-eat salads and minimally processed food products by several days compared to untreated controls, while maintaining acceptable sensory attributes. Furthermore, by reducing microbiological contamination, SACP diminishes the necessity for chemical preservatives and packaging interventions in ready-to-eat foods, aligning with clean-label and sustainable processing trends [129].

**Fig. 3:**
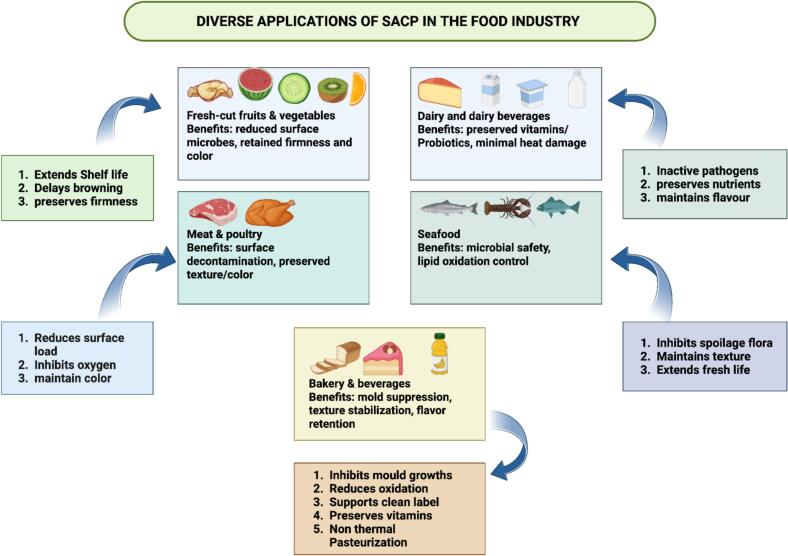
Diverse applications of sonication-assisted cold plasma in the food industry.

Fig. 3 shows the broad applications of SACP across different food segments, such as fresh-cut fruits and vegetables, dairy and dairy beverages, meat and poultry, seafood, bakery, and beverages. Each category derived a variety of benefits from SACP treatment, including improvement in shelf life, reduction of microbial population, and enhancement of nutritional and organoleptic quality, including proteolysis, as well as inhibition/spoilage organism growth and lipid oxidation controlling ability, proving it to be a non-thermal/non-additive clean-label technology to preserve food integrity in terms of extending its safety and quality.

## 6 Challenges and limitations

SACP can offer excellent benefits in food preservation; however, some issues must be addressed before it can be widely used in the food industry. SACP may be derived from process optimization, product variation, equipment design, regulatory agreements, or customer reception [66]. Addressing these issues is critical to harnessing the power of this disruptive nonthermal technology. Complications and diversity in the food matrix are among these challenges. Foodstuffs have various compositions, surface finishes, and moisture levels, which can influence SACP. However, porous and non-homogeneous products, such as bakery products, are not similar to liquid systems, such as milk or juices [71]. It is difficult to control parameters such as the exposure time, ultrasound intensity, and plasma discharge mode of the gas composition for every product. Without standardization, it is challenging to produce uniform results for various types of food.

The design and scale-up of the equipment are also of paramount importance. The majority of studies to date on SACP have been carried out at the laboratory or pilot scale, and the technical and economic challenges to transferring it to industrial-scale operation can be significant. For large-scale applications and uniform exposure of food surfaces to reactive species without excessive use of resources, this method should be efficient and economical [72]. The adaptation of SACP to current food processing and packaging lines would also require technological changes and capital investment, which may retard acceptability by industry. Regulatory and safety issues also impact the future trajectory of the SACP. This technology is not a visible residue and appears to be environmentally compatible; however, a systematic study about its safety and efficacy should precede the approval by food safety authorities (FDA, EFSA, or FSSAI) [130]. Regulatory clarity around plasma-treated foods is still emerging, and without a framework in place, commercial uptake might be limited.

## 7 Future perspectives

The application of SACP in food preservation has great potential, and a number of developing opportunities could make it more optimal, sustainable, and accepted by consumers. One of the main SACP trends is the progress in combination with smart packaging devices and biosensors [70]. Combined with smart packaging materials, nonthermal plasma processes contribute to the capability of online monitoring of changes in microbial load, pH, or spoilage data for dynamic control of food safety and shelf life. Such an integration would significantly reduce food waste and improve supply chain management. Another interesting approach involves the combination of SACP with natural antimicrobials (e.g., essential oils, plant extracts, and biopolymer coatings) [131]. These simultaneous applications could increase antimicrobial activity and have a greater reduction in microbial growth, with no adverse effect on the sensory and nutritional quality of food products.

Specifically, the penetration and bio-efficiency of bioactive compounds may be enhanced by plasma treatment, which in turn would result in multiple effects in the preservation aspect, which agrees with the clean label as well as minimal processing food trends. The SACP is the basis for personalized nutrition and functional foods. Due to the gentle preservation of bioactive ingredients and probiotics, as well as micronutrients by SACP, the produced foods could help to fit custom-made foods according to dietetic demand or health objectives [132]. This is in line with the increase in the demand for nutritionally enhanced/convenient functional foods by consumers.

## 8 Conclusions

SACP is a novel and versatile technology that can be introduced into modern food preservation processes. SACP has multiple effects on microbial inactivation, enzyme control, and quality preservation owing to the cooperative mechanism of ultrasound and reactive species generated by CP. This dual action has been found to extend the shelf life of different groups of food products, including fresh produce (fruits and vegetables), dairy, meat and poultry, seafood, bakery goods, beverages, and convenience foods, while preserving their sensory quality, nutritional value, and functionality. The main benefit of SACP is that it is nonthermal, has low nutrient loss, and minimizes the loss of color, texture, and flavor, in addition to reducing the application of synthetic preservatives. In vegetables, it delays enzymatic browning and microbial growth; in dairy/beverage, it safeguards probiotics, vitamins, and antioxidants; in meats/seafoods/poultry applications, it reduces spoilage and oxidative deterioration; and bakery or ready-to-eat food items inhibit the staling/mold forms. Together, these advantages solve critical food safety and shelf-life challenges and meet consumer interest in minimally processed “clean label” products. This procedure is a feasible, time-saving, and consumer-friendly alternative to conventional thermal or chemical preservation. This would contribute to industry needs and sustainability across the globe (food security, food loss, and waste), which is a function of postharvest cold chain management. With more studies, technological advances, and larger governmental acceptance, SACP can be considered a mainstream food preservation application that offers an all-inclusive the only promising solution for product shelf-life extension with the preservation of nutrition and sensory attributes.

## CRediT authorship contribution statement

**Vinay Kumar Pandey:** Writing – review & editing, Writing – original draft, Conceptualization. **Pinku Chandra Nath:** Writing – review & editing, Methodology. **Shailendra Thapliyal:** Writing – review & editing, Data curation. **Ayaz Mukarram Shaikh:** Writing – review & editing, Investigation, Conceptualization.

## Funding

The funding is supported by the University of Debrecen Program for Scientific Publication.

## Declaration of competing interest

The authors declare that they have no known competing financial interests or personal relationships that could have appeared to influence the work reported in this paper.
